# The anatomy of electronic patient record ethics: a framework to guide design, development, implementation, and use

**DOI:** 10.1186/s12910-021-00574-x

**Published:** 2021-02-04

**Authors:** Tim Jacquemard, Colin P. Doherty, Mary B. Fitzsimons

**Affiliations:** 1 FutureNeuro, the SFI Research Centre for Chronic and Rare Neurological Diseases, RCSI, 123 Stephen’s Green, Dublin 2, Ireland; 2 St. James’s Hospital, James’s Street, Dublin 8, Ireland; 3 Trinity College Dublin, Dublin 2, College Green, Ireland

**Keywords:** Electronic patient records, Electronic health records, Framework, Ethics, Electronic medical records, eHealth, Digitalisation

## Abstract

**Background:**

This manuscript presents a framework to guide the identification and assessment of ethical opportunities and challenges associated with electronic patient records (EPR). The framework is intended to support designers, software engineers, health service managers, and end-users to realise a responsible, robust and reliable EPR-enabled healthcare system that delivers safe, quality assured, value conscious care.

**Methods:**

Development of the EPR applied ethics framework was preceded by a scoping review which mapped the literature related to the ethics of EPR technology. The underlying assumption behind the framework presented in this manuscript is that ethical values can inform all stages of the EPR-lifecycle from design, through development, implementation, and practical application.

**Results:**

The framework is divided into two parts: context and core functions. The first part ‘context’ entails clarifying: the purpose(s) within which the EPR exists or will exist; the interested parties and their relationships; and the regulatory, codes of professional conduct and organisational policy frame of reference. Understanding the context is required before addressing the second part of the framework which focuses on EPR ‘core functions’ of data collection, data access, and digitally-enabled healthcare.

**Conclusions:**

The primary objective of the EPR Applied Ethics Framework is to help identify and create value and benefits rather than to merely prevent risks. It should therefore be used to steer an EPR project to success rather than be seen as a set of inhibitory rules. The framework is adaptable to a wide range of EPR categories and can cater for new and evolving EPR-enabled healthcare priorities. It is therefore an iterative tool that should be revisited as new EPR-related state-of-affairs, capabilities or activities emerge.

## Background

The increasing digitalisation of healthcare raises a range of ethical opportunities and challenges [1]. Digital healthcare can simultaneously advance and pose risks to ethical values such as patient autonomy, privacy and confidentiality and individual well-being. Understanding how it can promote or be at odds with ethical values is fundamental to accomplishing responsible digital healthcare. Applied Ethics is a practical approach to identifying and examining ethical concerns related to real world actions and practices [2]. This manuscript presents a framework to guide the discovery and assessment of ethical concerns associated with electronic patient records (EPR), which are a key component of healthcare digitalisation.

An EPR is a digital repository used to collect, store and display information regarding an individual’s medical history. EPRs support clinical care and health service administration and can often be used for secondary purposes such as research or billing in countries with market-based medical reimbursement systems. Compared to the traditional paper-based healthcare record, an EPR presents increased technical capability. Personal health information can be duplicated, shared, and queried with unprecedented speed and scale and therefore used in novel ways to benefit patient care, patient empowerment, efficiency of healthcare processes, and healthcare personnel (HCP) work satisfaction [3–5]. Clinical information is rendered more accessible when and where needed allowing better integration of healthcare services as the same healthcare record can be available to authorised HCPs at any location. Efficient interrogation of large volumes of individual or population data made possible with EPRs can support health service monitoring, evaluation, planning, public health and research [6–8]. These increased technological capabilities affect a broad range of direct and in-direct stakeholders including patients, their families and carers, HCPs, and third parties such as researchers or policy makers.

With the increased capabilities arising from EPRs comes new or alternative risks and potential for harm. Examples of negative impacts include clinician burnout related to EPR usage [9]; software defects resulting in incorrect drug prescriptions or instructions [10]; threats to privacy associated with poor database security [11]; copying and pasting other clinician's findings without clarifying its provenance [12]; templates design that lead to inaccurate information [13, 14]; promoted prescriptions that deviated from accepted medical standards [15]; and discriminatory algorithms stemming from biased EPR datasets [16]. In short, where EPR systems mediate healthcare delivery, regard for ethical values during all stages of the technology’s life-cycle from design, development, implementation through usage is paramount.

The framework described in this paper is designed to aid the identification of ethical challenges and opportunities associated with EPR technology. It is intended to support designers, software engineers, health service managers, and end-users to realise a responsible, robust and reliable EPR-enabled healthcare system that delivers safe, quality assured, value conscious care. The framework can be understood as an ‘ethical tool’ that guides “debates and deliberative structures for a systematic engagement with ethical issues” related to EPR technology in practice [17]. Development of the framework is based on a consolidation of EPR-related ethical challenges and opportunities debated in the literature that can inform decisions at every stage (design, development, implementation and use) of the EPR life-cycle [18]. While the framework offers an ethical tool, it should not be considered to end all philosophical deliberation of EPR implications. For example, the varied perspective of different users of the tool may lead to mixed interpretation of ethical issues. In this regard, multiple ethical tools can be used in parallel, as ethical tools can complement each other and form a “toolbox” [17].

## Methods

Development of the EPR applied ethics framework was preceded by a scoping review which mapped the literature related to the ethics of EPR technology [18]*.* That review identified a range of ethical values which were clustered into: privacy, autonomy, beneficence, human relationships and responsibility. In addition, it attributed responsibilities and duties to stakeholders including patient obligation to provide accurate information and clinician personal behaviours regarding correct documentation of patient records. To develop the framework, the same body of literature was re-examined to determine ethical challenges and opportunities debated in the literature in relation to characteristics across the EPR lifecycle from design, through development, implementation and subsequent use of the technology. In a series of repeat sessions, a multidisciplinary team consisting of an ethicist (TJ), senior hospital-based physician (CD), and an eHealth expert (MF) came together to discuss and reach consensus on interpretation of the literature. Discussions were guided by the assumption that ethical values shape the key components of the design, development, implementation, and use of EPRs.

The concept that decisions made at every stage of a system’s life-cycle can have ethically relevant implications is widely accepted within technology ethics [19–21]. Ethical opportunities or challenges occur when the technology supports or conflicts with ethical values. With EPR technology, these can be addressed through concrete and specific instructions, including EPR design requirements, codes of conduct regarding safe use of the system, and standard operating procedures. To illustrate, patient autonomy is an ethical value worth protecting. A related opportunity is to allow patients obtain a greater understanding of the personal health information held about them in an EPR. Specific instructions detail how this opportunity can be operationalised safely and in a manner that is understandable to the patient. The elements of the applied ethics framework described below emerged from considering how EPR technology either upholds or is incompatible with ethical values.

## Results

**Fig. 1:**
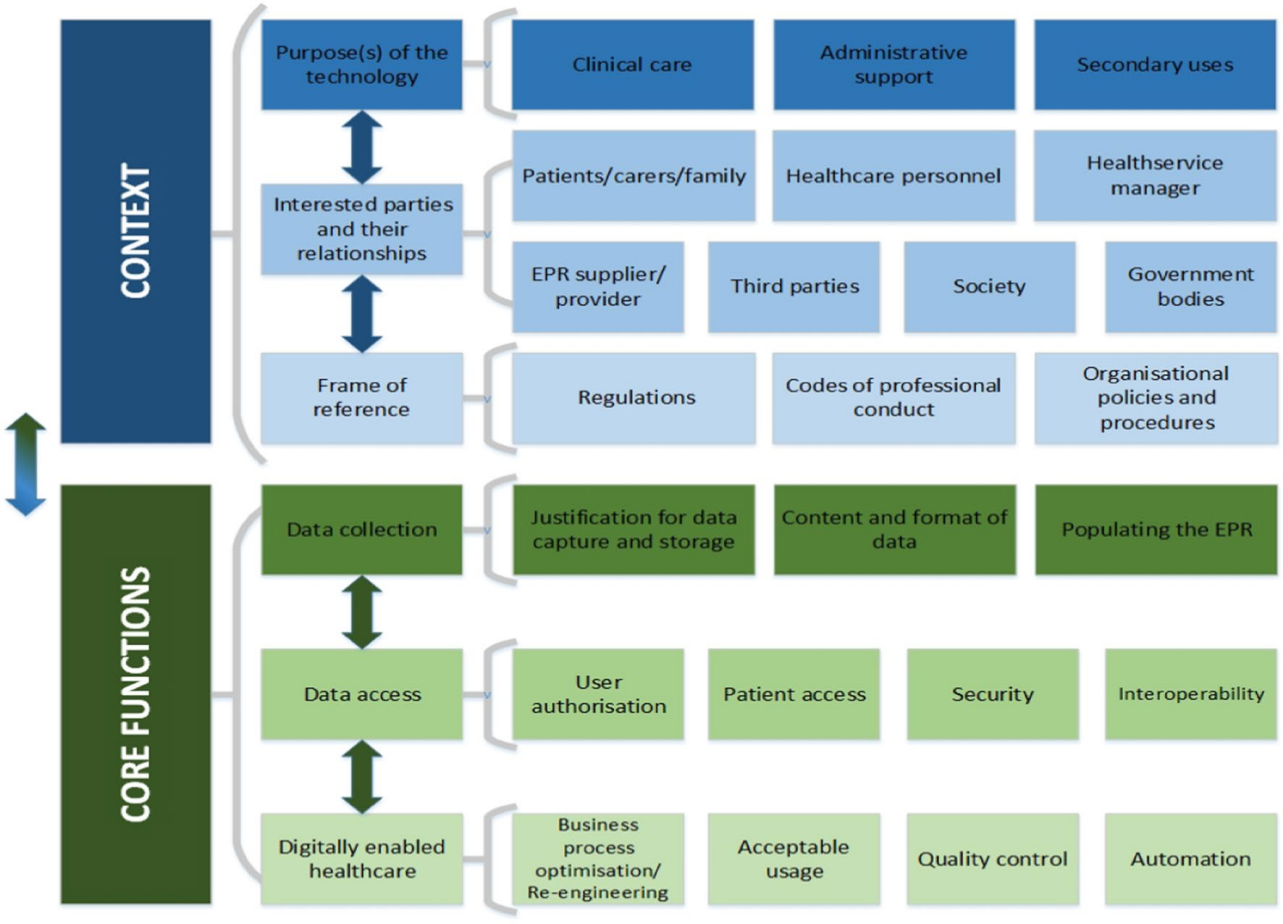
Electronic patient record (EPR) applied ethics framework

The resultant applied ethics framework aims to support identification and management of EPR-related ethical challenges and opportunities. It has two main sections, Context and Core functions, each of which has been divided into three categories which in turn have a number of attributes (Fig. 1). To apply the framework, an EPR of interest is assessed against each of its elements (sections, categories, and attributes) in order to identify any ethical considerations determine the associated benefits and/or risks, and put measures in place to appropriately address these issues. The framework should be applied at all phases of the EPR lifecycle to ensure robust requirements engineering and design, solid software development, reliable implementation, and safe use and evolution of the system.

While the framework user should start with examining the categories and attributes associated with the EPR context and then explore the elements related to its core functions, it must be noted that this is not a strict linear process as there is a strong interrelatedness between elements of the framework. For example, the “Format and Content” of “Data Collection” within the “Core Functions” section will have implications for the effectiveness of its “Secondary Uses” in the “Context” (Purpose(s) of the technology) section (e.g. analysis of unstructured data is more challenging than that of structured data). Use of the framework is therefore an iterative process. The outcome of each ethical assessment element can feedback to a previous element or feedforward to a subsequent element. Furthermore, the framework can be considered as a continuous quality improvement tool with, for example, repeated “Quality control” (Digitally enabled healthcare in Core Functions) or regular assessment of “Security” (Data access in Core Function) rather than at a single point in time.

In the following each element of the framework and its relevance is explained.

### Context

The first step in applying the framework is to clarify the context within which the EPR in question exists or will exist (Fig. 1). Understanding the context provides insight to the associated ethical challenges and opportunities. The process of establishing context should begin at concept stage (when the idea of introducing an EPR is being considered) and be reviewed as the EPR progresses along its life-cycle from requirements engineering and design specification through development and on to implementation and practical application. This will help ensure that the context remains constant or that any necessary changes to it are approved and appropriately addressed. Context comprises the *purpose(s)* for which the technology is used (e.g. clinical care, administration, secondary use), the *interested parties* involved in its design, development, implementation and use (e.g. IT developers, researchers, patients, health service administrators and managers and so forth), and the *frame of reference* (e.g. existing regulations, codes of conduct, and policies and procedures).

#### Purpose(s) of the technology

As it impacts almost all other elements of the framework, it is essential to begin with identifying the purpose(s) of the technology. For example, the *content and format* of data collected and stored in the EPR will be a function of its purpose. Ill-defined EPR purpose invites uncontrolled function and scope creep, creating complexity and ethical ambiguity [22, 23]. Without clarity of purpose(s), likelihood of EPR user and use error increases and can results in perceived failure of the technology. Opportunity costs may also arise as money spent on suboptimal EPR projects cannot be spent on other goods or services that can benefit patient care. Especially with public funding, ethics mandates a responsible use of finite resources [24]. Finally, clear determination of EPR purpose(s) informs the next steps in the framework application, namely ascertainment of the interested parties (“Interested parties and their relationships” section).

In the framework, EPR purposes are sorted into *clinical care*, *administration* and *secondary use* (Fig. 1). The ethical considerations and their level of importance will vary according to which of these EPR purposes are in play. An EPR user may use the system for different purposes simultaneously. Equally, different users can share an EPR purpose (e.g. a doctor and a nurse will both have a clinical care purpose) or have different purposes (e.g. health service manager may have an administrative support purpose while a researcher may be interested in the secondary use of data from the EPR).

In general, the use of EPRs to support clinical care is uncontroversial. By facilitating timely access to, and sharing of, information EPRs can become enablers of improved quality, safety and efficiency of healthcare [25]. Nevertheless, clarity regarding the scope of the clinical care purpose is essential to understanding who will be impacted by the EPR system and how it should be used in practice.

EPRs can also support healthcare management and administration functions such as billing, service performance reporting, patient administration systems (PAS), computerized physician order entry (CPOE) and so forth. While such utilities are fundamental to health service delivery, their integration into the EPR system should not negatively impact patient care. For example, administrative or managerial informational needs should not take precedence above the clinicians primary objective of safe patient care nor overburden clinicians with additional workload [26–28].

Secondary use of data from EPRs for purposes such as research, marketing, insurance, data brokering, and education impose additional ethical concerns and require greater justification. Such use can sometimes be justified by balancing the projected societal benefits against potential harms. While research based on EPR data may not directly benefit the data subjects, with minimal risk to them the research may lead to healthcare improvements for others [29]. Similarly, if the use of personal data for marketing or other commercial purposes fails to yield sufficient personal or societal benefits, secondary use may be considered undesirable [15, 30] (see also “Data collection” and “Data access” sections).

#### Interested parties and their relationships

In order to appropriately attribute rights, duties and responsibilities across its life-cycle, those individuals or groups who can either be affected by or affect the safe and ethical design, development, implementation, and use of the EPR must be identified. Furthermore, how these interested parties or stakeholders relate to each other and exert influence on the design, development, implementation, and use should also be considered. The framework (Fig. 1) suggests that EPR stakeholders range from individuals to groups and organisations and include patients [31], their families and/or informal care-partners [32–36], clinical personnel [37], health service managers [38], EPR supplier [39], society [29] and government bodies [40, 41].

To guide identification of interested parties, a number of questions should be asked. Examples include: whose personal data will be captured and stored in the EPR? Who can benefit from, or be harmed by the system? Who has or requires access to the EPR or (part of) its data? Who provides and maintains the EPR system? Who influences requirements engineering, design specification, implementation, and operational decisions about the uses and functionalities of the system or its data?

While the patient is the apparent principal EPR data subject, personal data interests of others can also be affected and therefore require careful consideration. In recording and storing personal health data about a specific patient, EPRs may also capture information about others in close proximity to the patient (e.g. genomic data, family history). Similarly, through audit trails, EPRs capture data about healthcare providers who use the system and can therefore reveal information about the HCP’s productivity which may be used to evaluate their performance.

Realising EPR benefits, such as the facilitation of information exchanges between different healthcare providers both within and between healthcare facilities, requires the ability to navigate the complexities of diverse informational needs and varied roles of different HCPs in delivering healthcare services [37]. Detailed understanding of these roles, their inter-relationships, and when and where they are executed is key to informing the design, development and implementation of the EPR and consequently achieving an ethically robust EPR-enabled health service delivery.

In its duty of care to patients and accountability to the funder, health service management has an essential stake in the EPR domain. Policies and standard operating procedures regarding, for example, rules for using the EPR and technical security measures to prevent data breaches must be operationalised by personnel responsible for day-to-day management of the system [42, 43].

The role of the EPR provider/vendor is critical. Confidence in the supplied product and its on-going maintenance requires a collaborative relationship between the healthcare organisation and provider. Without this, the EPR provider/vendor may exert undue influence on the shape and use of the technology. For example, vendor lock-in may ensue where switching costs are prohibitive, the transfer of data is too difficult, or when there are no available alternatives to the particular EPR system [1]. Decisions about proprietary rights, including ownership of data stored in the EPR [44, 45], are therefore important, as well as agreements around system support. A vendor who stops support for an application can leave the client with an unsafe system.

As noted previously, a variety of third parties may have interest in the data held in an EPR e.g. researchers, auditors, and marketers. As their interests will present particular ethical challenges and opportunities, it is important to consider these stakeholders early in the establishment of the EPR context so that they can be appropriately addressed across all stages of the EPR life-cycle. For example, commercially biased EPR-based clinical decision support tools, and perverse incentives to employ them, may influence prescribing behaviours in clinicians that may be detrimental to safe patient care [15].

Government bodies play a role in creation of a trustworthy environment for the use of personal health data and have a stake in how EPRs can enable improved quality and efficiency of healthcare [46]. They also have a responsibility to ensure prudent use of tax-payers money to fund EPR procurement as well as providing the necessary legislative basis for technology-enabled healthcare. Similarly, wider society has a stake in the ethical impact of EPRs. For example, the large volumes of data contained within EPRs can enable vital public health research [29]. Citizens have an interest in and expectation that such use of their health data is safe and ethical.

#### Frame of reference

While the ‘purpose’ and ‘interested parties’ elements address “what” and “who” contextual considerations, the frame of reference deals with “how” EPR-enabled healthcare is embedded within existing regulations, professional codes of conduct and organisational policies.

Regulations can support the establishment of a trustworthy environment for patients and healthcare organisations to capture and share personal health data. For example, principles of privacy by design, data minimisation, transparency and so forth will guide EPR requirements engineering and design specification, software development, and standard operating procedures for EPR use that are required to safeguard the rights and freedoms of data subjects [47]. However, regulation regarding the management and use of healthcare data is not standardised across all jurisdictions with some offering less protection of data subjects (patients) than others [48–50]. In some instances, legal requirements can actually jeopardise efforts to protect confidentiality as seen in China where medical information used to combat COVID-19 is now being used by local governments for different purposes [51–53]. Codes of professional conduct however, oblige healthcare professionals to maintain reliable records of engagement with their patients and to do so in a manner that respects confidentiality and guards against any unauthorised or accidental disclosures of patient information [51, 54, 55].

Healthcare organisations both at the level of the wider system and discrete healthcare facilities adopt policies and procedures to ensure legal and regulatory compliance, and guide best practice for its day to day operations [56, 57]. In establishing EPR context, relevant policies and procedures such as *inter alia* recommendations for healthcare records management should be considered. Where such policies and procedures were formulated to guide the use of traditional paper-based medical records, they may need updating to appropriately address the extended capabilities offered by EPR systems [40, 41].

### Core functions

Despite their many permutations, EPR systems share three core functions (Fig. 1). Firstly, they facilitate *Data Collection (capture and storage)* primarily about patients but also about family members and healthcare providers (see “Interested parties and their relationships” section): even more so than paper records, the design of an EPR determines which information can be captured and stored in a medical record. Secondly, they provide *Data Access* to a range of interested parties. EPRs facilitate access to health information for HCPs, administrative personnel and other parties. Finally, EPRs make possible *digitally enabled healthcare.* These core functions present a range of ethical challenges and opportunities that necessitate careful consideration of the interaction between an ensemble of people, processes and the technology.

#### Data collection

Key attributes of data collection have ethical implications and relate to: justification for its capture and storage; its content and format; and matters associated with populating the EPR (Fig. 1). In turn each of these attributes will be influenced by the EPR context and will determine who can or should be approved to use the EPR.

To justify the capture and storage of personal data in an EPR, clinical benefits for patients should be the guiding aim. Clinical benefits must significantly outweigh the risks to patient care both in probability and magnitude [24]. In terms of clinical or administrative purposes, so long as the appropriate safeguards are in place, capture and storage of data in an EPR may be considered justifiable in so far as they enable health service providers fulfil their contractual responsibility to service consumers. Where substantial EPR-related benefits and minimal harms exist, unless it is mandatory, waiving patient consent to capture and store their information may be pragmatic as, the consenting procedures can be resource intensive, the HCP-patient power-balance may interfere with the process, and in emergency situations, patients may (temporarily) lack capacity to provide consent [58–60]. However, where the inclusion of particular data types in the EPR poses a potential for harm [59–62], consent may be necessary to ensure the benefit-harm ratio is acceptable to the patient or their carer. An example can be seen in dermatological photography of the genital area or the entire body where increased privacy risks may arise [61].

Meanwhile, with collection of data in the EPR for secondary purposes such as research, the benefit-harm ratio often becomes more speculative so that respect for patient autonomy becomes more important [62, 63]. In these cases, the patient’s informed and voluntary decision about becoming the subject of an EPR system is indicated. Whatever the circumstances (clinical or secondary), the collection of their personnel health data should be transparent to data subjects so that they can exercise their rights in relation to its use if and when needed.

Healthcare involves a wide range of data types ranging from alpha-numeric, images, bioelectric signals and so forth [64]. The purpose of the EPR will determine its data content in terms of the type(s) of data and number of data fields or data tables captured and stored in the system. Data may be formatted in either structured data or unstructured free-text fields. Design of data collection and display interfaces must ensure that an EPR has no harmful effects on the quality and efficiency of patient care nor on the administrative burden or work satisfaction of the end-user of the EPR [26–28]. As a combination of both standardised data and patient nuances are essential for safe clinical care, EPR functionality should facilitate an appropriate balance between structured data and free-text [31, 65–68]. Meanwhile, structured data may be preferred by administrators and managers as it is more amenable to analysis and consequently can inform health service performance monitoring and evaluation. However, the format and content of data desired by administrators should not override informational needs of healthcare teams or patients [26, 27, 31].

Populating the EPR relates to how and where data is entered into the EPR and by whom. This is an important step, as the quality of record keeping can affect the quality of care and the well-being of patients. The process must lead to high quality EPR data as mistakes or omissions can lead to medical errors [69–76] negatively affecting the well-being of patients. Therefore, clinician personal behaviours, such as honesty, accuracy, and conscientiousness in completing patient records and entering data to the EPR is fundamental to the quality and safety of care. As populating the EPR can often be seen as time-consuming, shortcuts or workarounds which can increase risk are sometimes adopted to reduce the burden. For example, copy and paste functionality used to accelerate data entry may result in incorrect data, and abundance of redundant material in an individual’s EPR [28, 77–79] and thereby negatively affecting the utility of the medical record and possibly harming care. In some settings, professional scribes have been hired to assist with populating the EPR. However, this practice gives rise to confidentiality and privacy concerns, as it introduces a third party [80]. Additionally, capturing data for subsequent secondary usage can have other unintended negative consequences when it results in pressure on HCPs to populate EPR data fields that are not directly related to the patient’s clinical needs. Likewise, use of EPRs to support administrative purposes or health service financial management may lead to *upcoding* if the HCP is influenced to overstate diagnoses for monetary gain or commercial reasons [15, 81, 82]. To promote best practice, only authorised and trained users should enter data into the EPR and perverse incentives should be avoided.

#### Data access

The ease and promptness of data sharing and exchange made possible by EPRs enables access to and distribution of health data for a variety of clinical, administrative and secondary uses. For example, it can facilitate delivery of integrated health services and improved continuity of patient care, by providing HCPs with timely access to accurate information required to deliver healthcare services [67, 83, 84]. However, in the same way that capturing and recording data in the EPR must be justified, subsequent access to it must also validated and limited only to those who have ethically sound grounds to do so. Such grounds will depend on EPR context (purpose, stakeholder and frame of reference) and must be based on balancing autonomy of the data subject (patient) against the benefits-risks ratio associated with sharing and exchanging their data.

In terms of clinical care, decisions regarding access to data held in an EPR may be guided by asking questions such as: which healthcare professionals/providers are part of the patient’s circle of clinical care? What patient data access do they require? What are the potential harms resulting from the distribution of this information and how can these be mitigated? When determining rules regarding access to EPR data for clinical purposes, a tension between the data needs of the HCP to fulfil their healthcare responsibilities and the patient’s ability to decide that sharing their record (or parts of their record) is in their best interests [31, 74, 85–97] must be addressed. However, as previously noted, the process of capturing patient consent can be challenging. In addition, placing the onus on patients to make decisions may diminish the value or completeness of the EPR data if they refuse to give access to certain HCPs or to include or share specific pieces of information. For those adopting EPR technology, this tension may be reduced by ensuring transparency regarding the access to and distribution of patient data thus allowing the patient some form of control.

Unless there is a reasonable expectation that access will lead to significantly serious harm to the their physical or mental health, patients should be able to obtain access to their personal information. Patient portals are a digital solution for such access that can facilitate a degree of patient control over the content and sharing of their personal healthcare information. However, some ethical challenges must be taken into account when giving patient’s access to their own record. Examples include: ensuring the patient receives information in an understandable format [70, 85, 98, 99] and is supported in interpreting EPR content such as results of clinical investigations [94]; and avoiding HCPs purposely not documenting information in the EPR for fear of evoking a negative reaction from the patient [28, 99]. Another example relates to carers having access to the EPR on the patient’s behalf. For example, when parents have access to their child’s record there may be privacy implications [32–36]. Similarly, confidentiality may be impacted when an estranged parent reads information about themselves in their child’s EPR [35].

Authorising access to data in the EPR for secondary purposes, such as public health or the advancement of scientific knowledge [29, 36, 41, 71, 73, 75, 100–107], requires specific rules. With minimal risks to the patients and significant societal benefits, such efforts may take place without requesting consent, for example if records-based research is considered to pose minimal risk and/or where consent is impractical to obtain [29]. However, careful scrutiny of benefits and harms associated with secondary EPR data use is essential. For example, a vendor may provide an EPR free of charge with an understanding that they may capitalise on access to patient data [108]. As a result, a healthcare organisation may experience an unacceptable loss of control over the functioning of the EPR and the full realisation of its benefits [87, 109].

Risks associated with the EPR relate to unauthorised access to the data stored in the system, whether through intrusion by hacking or login misuse, or through data sharing without appropriate agreements. To protect personal information from such unauthorised access requires a set of policies, procedures, staff training and technical infrastructure [110, 111]. Codes of conduct oblige healthcare professionals to behave in a manner that does not facilitate data breaches. For example, users must not share passwords to the EPR system nor leave the EPR screen open [42, 43, 112, 113]. HCP training in the safe and secure use of EPR system is imperative [110, 111].

Safety and security in terms of user authentication, data access, data storage and backup, and acceptable usage should be incorporated into the design of the EPR [36, 73, 114–120]. Features that strengthen security include audit trails and role-based access controls (RBAC). The former are a chronological record of who has had access to the EPR, what they have accessed and any updates they have made to the record. With RBAC, EPR users have certain permissions related to their function in the healthcare team. For example, clinical personnel or administrative personnel access will be limited to the records of those patients for which they have clinical responsibility or to those elements of individual patient’s records that are relevant to their role. Where information might carry stigma, such as in the case of mental illness, substance abuse, and sexual health [117, 121, 122], the importance of defining appropriate access restrictions to parts of a patient’s record increases.

When the EPR is used for secondary purposes such as to teach medical students or in the case of data analytics research, security can be promoted through de-identification of the dataset of interest (information that leads to directly or indirectly identifying patients is altered or removed). However, it should be noted that even with de-identification achieving a sufficient level of anonymisation can be challenging as, for example, linkage of two or more datasets can lead to re-identification of data subjects [32, 84, 90, 94, 95]..

Interoperability is the facility of different EPR systems to share patient information within and across organizational boundaries. It enables improved integration of healthcare services and continuity of patient care [123]. Achieving interoperability is not simple as it requires processes and data to be harmonised across different healthcare services and facilities [61, 124–126]. For example, individual unique identifiers are required so that records about one patient held in different EPRs can be safely and accurately matched [32]. Poor interoperability poses ethical concerns as inadequate data co-ordination may lead to error and impact patient safety or impair the full realisation of EPR benefits [71, 72].

#### Digitally-enabled healthcare

The manner in which EPR technology is used in practice requires careful consideration to ensure optimal digital healthcare business processes, acceptable usage of the EPR, continuous quality control of the system and to understand the consequences of EPR-related automation (Fig. 1).

EPR implementation is not simply about the technical artefact. Interaction between the people who will use the EPR, the processes it is expected to support and the technology must be analysed so that improvement opportunities are identified and acted upon. In clinical care, where an EPR is a poor fit for clinical practices a decrease in efficiency, a lower quality and safety of patient care, reduced HCP job satisfaction, and diminished integration across healthcare organisations [70, 114, 118, 124, 127–129] may result. Similarly, as a key enabler of remote or virtual care, requirements engineering must ensure that the EPR technology is carefully matched to the associated workflow and patients’ needs [130] as well as mechanisms to establish and maintain the patient-clinicians relationship [28, 131].

During a clinical encounter, EPRs can give rise to the HCP being preoccupied with the computer screen rather than interacting with the patient [28, 76]. This challenge is indicative of the importance of ergonomics and the design of physical healthcare spaces where the EPR will be used [132]. For example, the layout of clinic rooms should facilitate ease of use of the EPR in a manner that promotes inclusivity e.g. positioning the computer monitor so that it can be viewed by the patient as well as the clinician.

An organisation adopting EPR systems must put in place a set of rules or acceptable usage policy (AUP) that guides how the system should be used. The AUP should advise all EPR users of their responsibility to protect patient confidentiality, to acquire accurate and complete information, their obligation to comply with the policy, the right of the organisation to monitor compliance [44, 103, 116, 133–136] and transparency requirements related to utilising patient data for secondary purposes [137]. AUPs should be formulated to help educate and support all EPR users including for example researchers and students [116, 138, 139]. Likewise, to ensure the AUP works in practice and does not disrupt workflows [140], representative EPR users with relevant expertise should be involved in devising it [134, 141, 142]. As previously noted, codes of professional conduct and employment contracts also inform acceptable usage of EPRs in practice and where necessary, these should be amended to address the new and emerging capabilities made possible with EPR technology [54].

To ensure that the expected benefits are realised and continuously improved, and that no unintended consequences arise, a process of EPR quality control should be implemented [143, 144]. Regular evaluation of benefits and harms can be used to monitor how well EPR-enabled activities are working, to identify opportunities for improvement, and where necessary realign priorities. For instance, if administrative uses of the EPRs negatively impact patient care, processes or priorities can be adjusted [26, 145]. Continuous monitoring can also highlight any unfair distribution of EPR related benefits across patient populations. For example, such inequities might arise due to a digital divide [74], unequal access [146], cultural diversity [36], characteristics or socio-economic status of patients [28, 74, 87], or the clinical condition [147]. Furthermore, as the cost of EPRs should not negatively impact patient services [73, 93, 148], regular review should allow any disproportionate outlay to be seen.

Third parties integrating health data from EPRs into their processes need to be aware of the limitations of the information captured. For example, biases in datasets can affect the outcome of research [72, 73, 75, 149, 150]. Biases can occur when the EPR system has been configured to suit a particular patient population, health concerns that are more prevalent in a particular region, administrative needs or regional medical guidelines [151] or when EPR-based clinical decision support results in drug prescribing behaviours that are influenced by commercial interests rather than clinical needs [15]. In addition, EPRs should not be considered the only relevant source of information for clinical care. Patients, carers or other HCPs may contribute information outside the EPR and their expertise should be considered [28, 131, 152].

The fusion of artificial intelligence (AI) such as machine learning with EPRs has the potential to automate certain parts of data capture, data distribution and communication of diagnoses to patients [153]. For example, chatbots that simulate human conversation, may allow users to access medical information [154]. Such automation may amplify existing ethical challenges and trigger new ethical questions. In their development, AI tools must be trained to conduct a desired task. Such training is based on large datasets containing personal health information of many patients that are sourced from EPRs. Sharing such datasets with AI algorithm developers/vendors is not without significant privacy implications. Furthermore, if biases exist in the training dataset then partiality may occur with the subsequent use of the AI tool [151, 155]. Additionally, automation can muddle responsibilities as clinicians who use AI tools to support clinical decision-making may need to weigh their own judgements against those of an algorithm. If digital avatars [156] are introduced to replace certain HCP tasks, face-to-face patient-clinician encounters are impacted, and critical data-entry errors may not be readily identified. Finally, AI algorithms may lack transparency so that the factors involved in their performance are not understandable to people who use, regulate, or are affected by the EPR system. The lack of transparency is a concern if algorithms are designed to promote commercial interests rather than aim to optimise clinical care [15].

## Discussion

Timely and efficient information sharing and exchange made possible through digitalisation promises to link healthcare services to healthcare constituencies (patients and healthcare providers at any location) thereby facilitating connected health and patient-centred care. EPRs are a foundational element of digital healthcare. However, safely and ethically embedding EPRs in the healthcare pathway involves an ensemble of factors that warrant careful consideration. This paper presents an applied ethics framework that may be used to guide decisions across all stages of the EPR life-cycle from requirements engineering and design specification, through development, implementation and on to practical application. The ultimate aim of the framework is to promote EPR-related practices that reap ethical opportunities while also addressing ethical challenges. Its development was based on a prior review of an extensive body of literature debating EPR-related ethical considerations and their determinants [14].

EPRs offer new capabilities that are unachievable with the traditional paper-based medical record. With EPRs, the same patient information can be available to all authorised healthcare providers regardless of their geographical location, multiple users can have simultaneous access, and large volumes of data are readily interrogated and analysed. As such, EPRs are widely acknowledged as central to the aspirations of health service modernisation which aim to: deal with burgeoning demands being placed on healthcare systems; move away from simply treating illness to promoting health and well-being; and develop new models of integrated care that is delivered in the most appropriate setting for the patient [3–5]. Consequently, a responsible design, development, implementation, and use of EPRs necessitates consideration of relevant moral norms to guide digital transformation of health services. For example, a re-appraisal of actors involved in the care process is called for when intra- and inter-organisational multidisciplinary patient care is enabled with EPR technology. Similarly, EPR-enabled data analytics may require greater understanding of third party interests such as public health researchers or IT developers, as well as their input as appropriate across various stages of the technology life-cycle.

The presented EPR applied ethics framework was developed by considering a broad range of issues of ethical interest that can inform all phases of the EPR life-cycle in order to achieve desirable EPR-based outcomes and minimise or eliminate any negative impacts. In this regard, the framework differs from, but may be complemented by, legal or regulatory obligations relevant to EPR adoption such as the European Union’s (EU) General Data Protection Regulations (GDPR) [47] or Health Insurance Portability and Accountability Act (HIPAA) [157] in the US. For example, the data protection impact assessment (DPIA), condition of GDPR supports concepts of privacy by design together with identification and minimisation of data protection risks. Hence, while the legally mandated DPIA focuses on data privacy and protection, the framework illustrated in this paper consolidates a broader range of ethical issues of interest in the EPR domain. Nevertheless, similar to how an EPR DPIA is documented and regularly reviewed and updated to reflect any data processing changes, the EPR ethics framework should be a living document, which is updated as necessary to reflect new state-of-affairs, capabilities or activities.

The primary objective of the EPR Applied Ethics Framework is to identify and create value and other benefits rather than to merely prevent risks. It should therefore be used to steer an EPR project to success rather than be seen as a set of inhibitory rules. The framework has some similarities to risk management employed by organisations to identify and mitigate threats to its function. The framework can augment a healthcare organisations risk management by, for example, preventing investments into sub-optimal EPR technology, and by guiding identification and control of risks arising from unethical EPR-related behaviour. Therefore, early incorporation of an applied ethics approach can help deliver patient safety, healthcare quality as well as economic benefits [19].

Although it has no legal standing nor associated direct financial penalties, the EPR applied ethics framework can influence various actors in bringing about value and benefits. Besides its normative value and peoples’ intrinsic desire to behave ethically, the framework has instrumental value. Failing to attend to ethical considerations can be socially costly resulting in, for example, clinicians being demotivated, healthcare personnel becoming less effective, and a decrease in the quality [9] and trust in the integrity of patient care [15]. Furthermore, financial implications may give rise to: funding being directed towards technologies that lack the support needed to make the desired impact [158]; vendor bargaining power that leads to a decrease in service quality and an increase in price; and clinical practices that are more costly than necessary. Designers, software engineers, vendors, end-users and other relevant actors can be guided by the framework to embed these and other ethical considerations into every stage of the EPR life-cycle.

Use of the framework may be motivated by different stakeholders’ desire to act responsibly and to maintain or enhance their reputation. Designers and developers want to ensure that their EPR system is robust, reliable and does not cause harm. Vendors want a product that meets the needs of their customers and develops a positive EPR market identity for their organisation. End-users want an EPR that can facilitate safe and effective healthcare service delivery. Even when there is a perceived or actual gain for one particular stakeholder through their engagement in, for example, “perverse incentives” or “vendor lock-in” practices, other stakeholders can use the framework to assess and address matters. For example, purchasers of an off the shelf EPR may require vendors to attest that their technologies are not influenced improperly by commercial interests. Similarly, concerns around EPR functionality or “vendor lock-in” may be mitigated by demanding that interoperability and data portability be designed and developed into the system.

The framework has the flexibility to deal with new and emerging EPR-related conditions. For example, the current COVID-19 pandemic has heightened an interest in the role of EPRs to support public health and epidemiological research, and delivery of remote/virtual healthcare. Managing a pandemic requires accurate and quick access to relevant health information. For example, in the UK, a unified dataset allowed rapid interrogation of health information of 17 million people to determine risk factors associated with death from COVID-19 [6]. In many other countries there is neither a comparable single, unfragmented dataset nor the technical infrastructure to query health information in an efficient and responsible way [159]. By showing what is at stake when timely data analytics capabilities are lacking, the pandemic may further guide how priorities for EPR data capture and sharing are established. However, health data analytics aspirations should not devalue the importance of relevant ethical values such as patient privacy or patient autonomy. Rather, the potential to use EPR-based data for public health purposes should be considered from the outset while primarily aiming to realise clinical benefit from the technology together with safeguarding patient autonomy, confidentiality and so forth.

### Limitations

Although the framework has been developed by a multidisciplinary team (TJ, CD, MF), its practical application has not yet been tested. However, a study is currently underway to examine the usability and utility of the framework from which guidelines for its operationalisation will emerge. The outcome of this study will be reported in due course. Additionally, as the framework is an ethical tool, it may fail to address legal concerns around EPRs. Nevertheless, it can complement relevant regulatory and legal considerations. Finally, the framework provides a broad and expansive overview of ethical challenges and opportunities associated with EPR technology across its life-cycle. The framework can therefore supplement other more specialised frameworks that discuss discrete challenges and opportunities in more detail thereby contributing to an EPR ethical toolbox.

## Conclusion

Responsible and ethical adoption of EPRs into the healthcare pathway involves a complex and interrelated ensemble of people, processes and technology. To support the management of this complexity, a framework based on literature regarding EPR-related ethical issues of interest has been developed. The framework presents a taxonomy of context and core function considerations that can help guide identification of EPR-related ethical challenges and opportunities. It should be applied across all stages of the EPR life-cycle from concept through to practical use in order to ensure the required measures are in place to achieve high-quality, safe and ethical EPR-enabled healthcare delivery.

## Data Availability

Not applicable.

## Abbreviations

AI: Artificial intelligence
AUP: Acceptable usage policy
DPIA: Data protection impact assessment
EPR: Electronic patient records
GDPR: General Data Protection Regulations
HCP: Healthcare personnel
HIPAA: Health Insurance Portability and Accountability Act
RBAC: Role-based access controls
